# Malaria prevalence metrics in low- and middle-income countries: an assessment of precision in nationally-representative surveys

**DOI:** 10.1186/s12936-017-2127-y

**Published:** 2017-11-21

**Authors:** Victor A. Alegana, Jim Wright, Claudio Bosco, Emelda A. Okiro, Peter M. Atkinson, Robert W. Snow, Andrew J. Tatem, Abdisalan M. Noor

**Affiliations:** 1 Geography and Environment, University of Southampton, Southampton, UK; 2 Flowminder Foundation, Stockholm, Sweden; 3 Faculty of Science and Technology, Lancaster University, Lancaster, UK; 4 School of Geography, Archaeology and Palaeoecology, Queen’s University Belfast, Belfast, BT7 1NN Northern Ireland UK; 5 Population Health Theme, Kenya Medical Research Institute-Wellcome Trust Research Programme, Nairobi, Kenya; 6 Centre for Tropical Medicine and Global Health, Nuffield Department of Clinical Medicine, University of Oxford, Oxford, OX3 7LJ UK; 7 World Health Organization, Geneva, Switzerland

**Keywords:** Indicators, Intra-class correlation, Malaria, Precision

## Abstract

**Background:**

One pillar to monitoring progress towards the Sustainable Development Goals is the investment in high quality data to strengthen the scientific basis for decision-making. At present, nationally-representative surveys are the main source of data for establishing a scientific evidence base, monitoring, and evaluation of health metrics. However, little is known about the optimal precisions of various population-level health and development indicators that remains unquantified in nationally-representative household surveys. Here, a retrospective analysis of the precision of prevalence from these surveys was conducted.

**Methods:**

Using malaria indicators, data were assembled in nine sub-Saharan African countries with at least two nationally-representative surveys. A Bayesian statistical model was used to estimate between- and within-cluster variability for fever and malaria prevalence, and insecticide-treated bed nets (ITNs) use in children under the age of 5 years. The intra-class correlation coefficient was estimated along with the optimal sample size for each indicator with associated uncertainty.

**Findings:**

Results suggest that the estimated sample sizes for the current nationally-representative surveys increases with declining malaria prevalence. Comparison between the actual sample size and the modelled estimate showed a requirement to increase the sample size for parasite prevalence by up to 77.7% (95% Bayesian credible intervals 74.7–79.4) for the 2015 Kenya MIS (estimated sample size of children 0–4 years 7218 [7099–7288]), and 54.1% [50.1–56.5] for the 2014–2015 Rwanda DHS (12,220 [11,950–12,410]).

**Conclusion:**

This study highlights the importance of defining indicator-relevant sample sizes to achieve the required precision in the current national surveys. While expanding the current surveys would need additional investment, the study highlights the need for improved approaches to cost effective sampling.

**Electronic supplementary material:**

The online version of this article (10.1186/s12936-017-2127-y) contains supplementary material, which is available to authorized users.

## Background

There is an increasing demand for high quality data and statistics to support decision-making and track progress towards the Sustainable Development Goals in low- and middle-income countries [1, 2]. This is not only important for scientific understanding of infectious disease epidemiology and health advocacy [3, 4], but is also useful for evaluating the impact of health investments [5, 6]. Since the mid-2000s there has been a renaissance in the use of nationally-representative cross-sectional household surveys [7], such as the Malaria Indicator Surveys (MIS) developed by the Roll Back Malaria Partnership [8], the Multiple Indicator Cluster Surveys [9], and the Demographic and Health Surveys (DHS) [10], as a source of public health intelligence data to track progress on uptake of some health interventions [11].

The above surveys are nationally-representative, and provide data on many indicators. For example, the DHS surveys include variables on population demographics, fertility in women, family planning, maternal and child health, and infectious diseases. The precision of these cross-sectional surveys is related to the sample size, budget, reference sampling indicator, the effect of indicator clustering, and the overall data quality affected by non-sampling errors [12–14]. In practice a reference indicator, usually an indicator for women of reproductive age (15–49 years) or indicator for pregnant women in the Kenya 2010 MIS, is used to determine sample size at design stage. Sample design is usually based on two-stage sampling design where primary sampling units (PSU) are first selected based on a complete list of census enumeration areas (EAs), followed by a random selection of households within the PSU [10]. While it is advantageous to collect data on many variables in a single cross-sectional survey, the overall optimal sample size requirements for all indicators are not always met and remain poorly defined due to difference between the reference sampling indicator and other indicators. Quantifying the precision in these indicators is important, not only in interpreting findings from the surveys, but also in designing future surveys.

Indicator precision can be quantified based on the effect of clustering as estimated via the intra-class correlation coefficient (ICC) [15, 16]. The ICC, in general, estimates the similarity of individual characteristics at a primary sampling unit [15]. It has previously been used in the design and analysis of cluster-randomized trials [17, 18]. Thus, a large estimate of ICC indicates greater homogeneity within the cluster and this requires only a small sample of households per cluster at the design stage. Conversely, a small intra-class correlation requires a large sample of households at cluster level, but fewer clusters nationally. ICC is related to the survey *design effect* due to clustering (loss of effectiveness) [19] commonly included in current national survey reports. Bayesian estimation of ICC has been examined in the design of clinical trials [20, 21]. The added value of the Bayesian approach includes: (i) such a framework is tractable for complex statistical models and various sources of uncertainty can be incorporated into the assessment of precision to aid comparability between indicators and surveys [22] and (ii) prior beliefs can be imposed on the cluster precision parameters which are updated upon observing the data. Such methods are used here for assessing survey effectiveness in sub-Saharan Africa.

In this research, measures of cluster-sample survey efficiency in recent cross-sectional surveys in nine countries in sub-Saharan Africa, were estimated. Although these surveys may differ in terms of implementation, the objective here was to assess the precision of three malaria-related indicators independently of the survey or the base sampling indicator. A retrospective analysis was conducted based on variability in malaria parasitaemia, and two other indicators, namely fever and the use of insecticide-treated nets (ITNs) for children under 5 years (0–4 years). For example, the effective sample sizes was estimated, retrospectively, for parasite prevalence independent of survey costs, and compared with the original survey estimates that were based on prospective analysis. Such estimates of clustering can be useful in interpretation of current survey data, and in designing and evaluating future surveys related to health and development. Moreover, costing modules can be implemented readily with these estimates to assess future financial needs.

## Methods

### Child morbidity indicators data

The main source of data was from nationally-representative population-based household surveys undertaken for estimating population health outcomes and risk factors. These included the demographic and health surveys (DHS), and the related disease-specific surveys, the Tanzania HIV/AIDS and malaria indicator survey and the standalone malaria indicator surveys (MIS). Data were assembled for the three indicators namely; prevalence of reported fever in the preceding 2 weeks, malaria test results from a rapid diagnostic test (RDT) based on a finger (or heel) prick blood sample, and use of ITNs. The three data variables were extracted only for children under the age of 5 years, along with survey dates, and geographical coordinates for each cluster-sample. Countries were selected if more than two nationally-representative household survey datasets were accessible, and data for the three indicators, including parasitaemia testing were available along with geographic coordinates at cluster level. Therefore, although data on the use of ITNs and prevalence of fever were available from earlier DHS and MIS surveys (i.e. since 2000), the incorporation of parasitaemia testing started in 2006 [23]. This resulted in nine countries that met the criteria where data were available and the coverage period of surveys ranged from 2007 to 2016 (*n* = 20 surveys, 5839 clusters). These were in east Africa (Kenya, Uganda, Tanzania, and Rwanda), southeast Africa (Madagascar, Malawi), and in west Africa (Nigeria, Liberia, and Senegal). 14 of these surveys were MIS surveys, five were DHS surveys (two in Rwanda, two in Senegal and one in Tanzania), and one was an HIV/AIDS and MIS survey in Tanzania. The average number of households per selected cluster varied from 18 households in the 2012 Tanzania survey to 36 households in the 2007 Kenya MIS survey. Data points were excluded if the measure of child health outcome was not present (i.e., if a child was not listed in the household or due to a child death). Missing data were imputed as NAs if a child was listed in the household with the response marked as “don’t know”, or missing the result of parasitaemia.

### Random spatial sampling of DHS clusters

Simple random spatial sampling at the cluster level, representing 30% of clusters, was drawn without replacement (i.e. using an unweighted simple random sample). Consequently, no survey cluster appeared twice in the respective samples. Sampling was performed in ARCGIS 10 using the spatial random sampling design tool based on a sampling seed. For Kenya and Rwanda, a further test sample was drawn randomly without replacement representing 20% (*n* greater than 30 clusters) and 40% of clusters stratified by Administrative level 1 (ADMIN 1). The 30% subset sample (and 20, 40% samples) was used to estimate prevalence under simple random sampling (SRS) for the three indicators of fever, ITN use, and malaria parasitaemia testing. Of the total 5839 clusters in the nine countries, only a few (*n* = 34; 0.6%) did not have geographic coordinates and, therefore, these were not used in spatial random sampling selection. An estimate of bias was, therefore, based on the difference between the Bayesian simulated posterior mean and the expected value under SRS. The remaining 70% sample was used for simulation via the Bayesian Markov chain Monte Carlo (MCMC) [24, 25] approach to generate posterior distributions for various measures of survey effectiveness. The objective of the MCMC algorithm was to obtain a stationary distribution with Monte Carlo integration used to approximate posterior expectations of the parameters of interest. The posterior samples were then used for inference. These included summary statistics of the posterior distribution of the indicator, between- and within-cluster posterior variance, the intra-class correlation coefficient (ICC), the survey design effect (*deff*), and posterior estimate of the effective sample size (ESS).

### Modelling household survey effectiveness parameters

Data were analysed by survey and by country, rather than pooling, such that there was no method induced correlation between surveys. The Bayesian hierarchical model was implemented via the MCMC approach in JAGS version 4.2.0 [26] and the *R2jags* package in R version 3.3.1 [27]. The proportion of total variability between- and within-clusters was estimated using the ICC(*ρ*). ICC was estimated assuming a binomial distribution for number of individuals examined at a cluster based on RDT result, prevalence of fever or use of ITN, adjusting for person age, and survey domain stratification by urban or rural setting. The survey design effect was then modelled in the same framework based on estimates of ICC and used to derive the optimal sample size based on the same number of clusters used in the original survey. For example, 200 clusters with an average cluster size of 36 households were used to simulate the effective sample size in the 2007 Kenya MIS. The model parameter convergence rate was evaluated using a combination of the Gelman-Rubin [28] and Raftery-Lewis methods [29]. For the former, a reduction factor of < 1.05 was used (the proposed threshold for detecting stationarity of a target posterior distribution in MCMC implementation). The latter provided estimates of burn-in and thinning factors given an accuracy of 0.005 and coverage probability of 0.95 (probability of the true values contained within predicted credible interval). Extended descriptions of the methods are provided in Additional file 1.

## Results

**Fig. 1:**
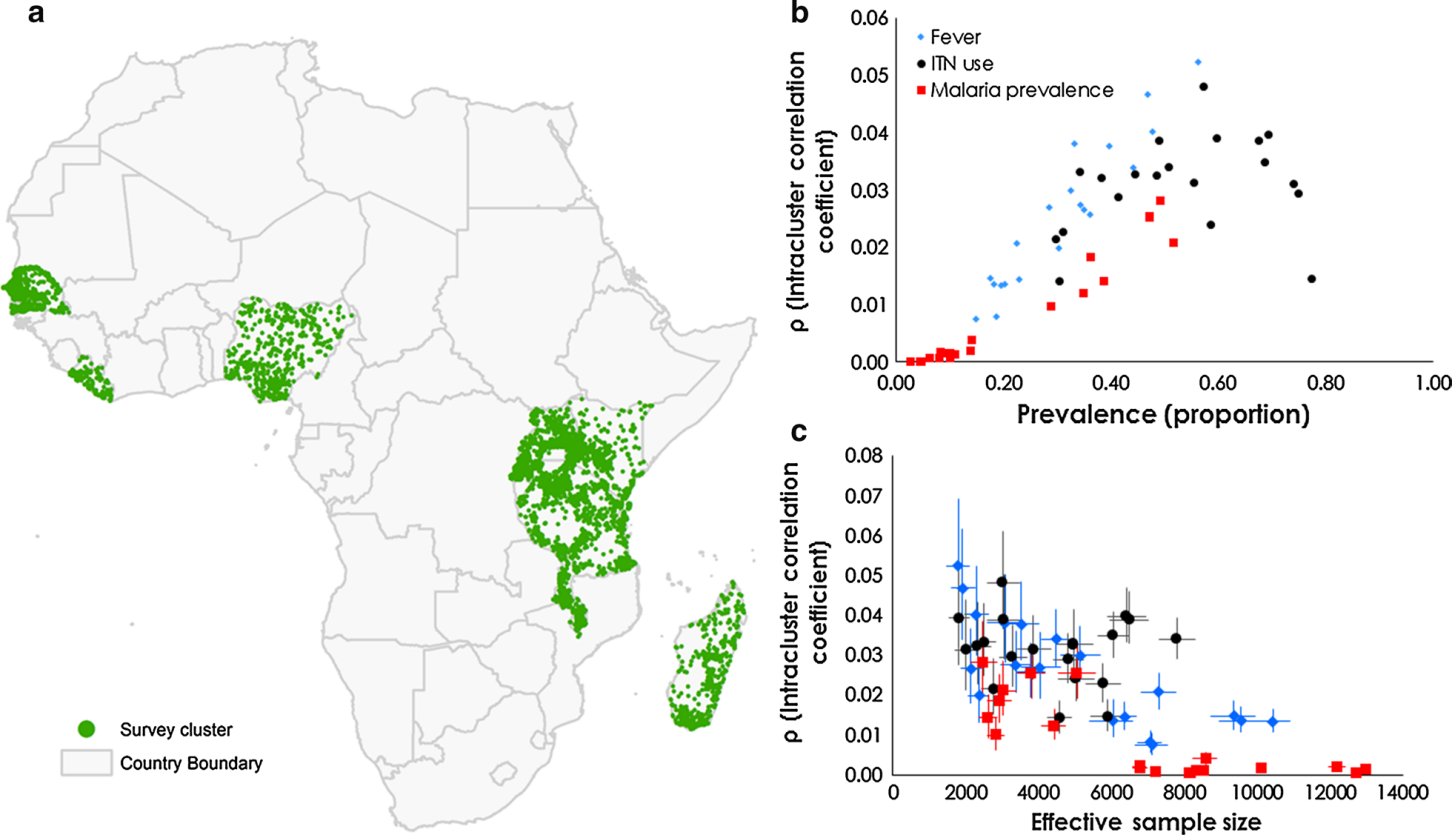
**a** The spatial distribution of clusters (*n* = 5839) in the 20 surveys in nine countries. **b** A scatterplot of the intra-class correlation coefficient and the modelled estimate of prevalence for the three child morbidity indicators [fever prevalence, ITN use and malaria prevalence based on rapid diagnostic testing (RDTs)]. Each data point in the scatter represents a survey. *ρ* refers to the variability between clusters and shows that surveys with low prevalence exhibit small between-cluster variance. **c** A scatterplot showing the decrease in *ρ* as Effective Sample Size increases with a Bayesian 95% credible interval

Table 1 shows the results for the 5839 clusters (134,399 children 0–4 years) split into the multiple data sources across nine countries, including estimated household survey effectiveness parameters for malaria prevalence only. For reasons of space, tables for the reported 14-day fever prevalence and the use of ITNs are included as Additional file 2: Table S1, Additional file 3: Table S2, respectively). Figure 1a shows the spatial distribution of these clusters in the nine countries. The Bayesian mean, median and 95% credible intervals (Crl) are presented along with estimates of ICC, *deff* and the ESS.

**Table 1:** Statistics relating to the prevalence of malaria parasitaemia from the nationally representative household surveys (the demographic health survey (DHS), the malaria indicator survey (MIS) and the HIV/AIDS and malaria indicator survey)

Country	Survey, year, and months	Number of survey clusters (number missing geographic coordinates)	Number of children under five	Simple spatial random sample (mean proportion)	Bias	Simulated proportion. Mean; median; (95% CrI)	Intra-class correlation coefficient (ICC). Mean; median; (95% CrI)	Design Effect. Mean (*hdeff*); median; (95% CrI)	Effective sample size (ESS). Mean; median; (95% CrI)	Percentage increase or decrease (95% CrI) in ESS compared to actual survey sample
Kenya	MIS 2007 June to July	200 (1)	3423	0.07	− 0.01	0.09; 0.09 (0.08 to 0.1)	0; 0 (0 to 0)	1.06; 1.06 (1.03 to 1.1)	6805; 6814 (6575 to 6983)	99.07 (92.08 to 104)
Kenya	MIS 2010 July to August	240 (8)	5104	0.14	0.00	0.14; 0.14 (0.13 to 0.14)	0; 0 (0 to 0)	1.06; 1.06 (1.04 to 1.09)	6788; 6797 (6584 to 6953)	33.17 (29 to 36.23)
Kenya	MIS 2015 July to August	246 (0)	4063	0.09	− 0.01	0.10; 0.10 (0.09 to 0.11)	0; 0 (0 to 0)	1.02; 1.02 (1.01 to 1.04)	7211; 7218 (7099 to 7288)	77.65 (74.72 to 79.37)
Liberia	MIS 2008 to 2009 December to March	150 (0)	4611	0.36	0.00	0.36; 0.36 (0.34 to 0.38)	0.02; 0.02 (0.01 to 0.03)	1.54; 1.54 (1.38 to 1.73)	2930; 2930 (2597 to 3254)	− 36.46 (− 43.68 to − 29.43)
Liberia	MIS 2011 September to December	150 (0)	3692	0.52	0.03	0.49; 0.49 (0.47 to 0.51)	0.03; 0.03 (0.02 to 0.04)	1.83; 1.82 (1.58 to 2.12)	2477; 2476 (2123 to 2843)	− 32.94 (− 42.5 to − 23)
Madagascar	MIS 2011 March to May	268 (1)	7138	0.06	0.00	0.06; 0.06 (0.05 to 0.07)	0; 0 (0 to 0)	1.03; 1.03 (1.02 to 1.04)	8349; 8354 (8234 to 8437)	17.04 (15.35 to 18.2)
Madagascar	MIS 2013 May to June	274 (0)	6288	0.07	− 0.01	0.08; 0.08 (0.07 to 0.09)	0; 0 (0 to 0)	1.03; 1.03 (1.02 to 1.04)	8538; 8544 (8414 to 8630)	35.88 (33.81 to 37.25)
Malawi	MIS 2012 March to April	140 (0)	2436	0.39	0.01	0.39; 0.39 (0.37 to 0.4)	0.01; 0.01 (0.01 to 0.02)	1.35; 1.34 (1.23 to 1.49)	2604; 2609 (2352 to 2857)	7.1 (− 3.45 to 17.28)
Malawi	MIS 2014 May to June	140 (0)	2249	0.32	0.03	0.29; 0.29 (0.27 to 0.31)	0.01; 0.01 (0.01 to 0.01)	1.24; 1.24 (1.15 to 1.35)	2827; 2831 (2592 to 3042)	25.88 (15.25 to 35.26)
Nigeria	MIS 2010 October to December	239 (0)	4950	0.47	0.00	0.47; 0.47 (0.46 to 0.49)	0.03;0.03 (0.02 to 0.03)	1.64; 1.64 (1.49 to 1.82)	3793; 3797 (3411 to 4182)	− 23.29 (− 31.09 to − 15.52)
Nigeria	MIS 2015 October to November	326 (4)	7016	0.44	− 0.03	0.47; 0.47 (0.46 to 0.49)	0.03; 0.03 (0.02 to 0.03)	1.62; 1.61 (1.47 to 1.79)	5059; 5063 (4541 to 5549)	− 27.84 (− 35.28 to − 20.91)
Rwanda	DHS 2010 to 2011 September to March	492 (0)	8963	0.02	0.00	0.02; 0.02 (0.02 to 0.03)	0; 0 (0 to 0)	1.01; 1.01 (1 to 1.01)	12720; 12730 (12,660 to 12,760)	42.03 (41.25 to 42.36)
Rwanda	DHS 2014 to 2015 November to April	492 (0)	7931	0.08	− 0.01	0.08; 0.08 (0.07 to 0.09)	0; 0 (0 to 0)	1.05; 1.05 (1.03 to 1.07)	12,210; 12,220 (11,950 to 12,410)	54.08 (50.67 to 56.47)
Senegal	MIS 2008 to 2009 November to February	320 (2)	16,156	0.12	− 0.02	0.14; 0.14 (0.13 to 0.15)	0; 0 (0 to 0.01)	1.12; 1.12 (1.08 to 1.17)	8590; 8599 (8231 to 8895)	− 46.78 (− 49.05 to − 44.94)
Senegal	DHS 2010 to 2011 October to May	391 (6)	13,334	0.03	− 0.01	0.04; 0.04 (0.04 to 0.05)	0; 0 (0 to 0)	1.01; 1.01 (1 to 1.01)	8161; 8163 (8120 to 8188)	− 38.78 (− 39.1 to − 38.59)
Senegal	DHS 2012 to 2013 September to June	200 (0)	7413	0.04	0.00	0.04; 0.04 (0.04 to 0.05)	0; 0 (0 to 0)	1.01; 1.01 (1 to 1.01)	8169; 8171 (8144 to 8189)	10.23 (9.86 to 10.47)
Tanzania	HIV/AIDS and MIS 2011 to 2012 December to May	583 (10)	9319	0.10	0.00	0.10; 0.10 (0.09 to 0.11)	0; 0 (0 to 0)	1.04; 1.04 (1.03 to 1.05)	10,130; 10,140 (10,010 to 10,230)	8.81 (7.41 to 9.78)
Tanzania	DHS 2015 to 2016 August to February	608 (0)	10,901	0.12	0.01	0.11; 0.11 (0.1 to 0.11)	0; 0 (0 to 0)	1.03; 1.03 (1.02 to 1.04)	13,000; 13,000 (12,880 to 13,110)	19.26 (18.15 to 20.26)
Uganda	MIS 2009 to 2010 November to February	170 (0)	4202	0.53	0.02	0.51; 0.51 (0.5 to 0.53)	0.02; 0.02 (0.02 to 0.03)	1.57; 1.57 (1.41 to 1.78)	3035; 3038 (2680 to 3366)	− 27.7 (− 36.22 to − 19.9)
Uganda	MIS 2014 to 2015 December to February	210 (2)	5210	0.33	− 0.02	0.35; 0.35 (0.33 to 0.36)	0.01; 0.01 (0.01 to 0.02)	1.33; 1.33 (1.24 to 1.44)	4425; 4424 (4074 to 4743)	− 15.09 (− 21.8 to − 8.96)

Data for prevalence of fever and use of insecticide treated bed nets from the same surveys is shown in the Additional file 2: Table S1 and Additional file 3: Table S2, respectively. In total, there were 134,399 children aged 0–4 years in 5839 clusters. The spatial distribution of these clusters in shown in Fig. 1a. Data includes surveys where the three indicators were simultaneously collected at the household level and in countries with more than two surveys. The mean, median and 95% credible intervals of the marginal posterior distribution from the Bayesian analysis are presented for measures of effectiveness: intra-class correlation coefficient (ICC), the estimated design effect (*hdeff*) and the effective sample size (ESS). The absolute bias is the difference in the means between the simple spatial random sample and the Bayesian model estimate. *ICC* intra-class correlation coefficient, *ESS* effective sample size; CrI Bayesian credible interval

There was little difference in absolute bias, across the three indicators, between prevalence estimated from a simple spatial random sampling scheme and prevalence estimated from the Bayesian parameter model adjusted for age. This was also the case when simple random spatial sampling represented only 20 or 40% of the clusters (Additional file 4: Table S3). The test for convergence (Gelman–Rubin test) was less than 1.05 for all the parameters monitored in the MCMC implementation. The Raftery–Lewis method showed that a minimum of 3746 iterations were required to achieve an accuracy of 0.005 at coverage probability of 0.95 for most indicators and a minimum of 10,510 iterations to achieve an accuracy of 0.0025 at coverage probability of 0.99. However, all the Bayesian model-based results are based on 55,000 iterations on two chains with a burn-in of 5,000 iterations and retaining every 50th iteration to produce a weakly dependent sample of size *n* = 1000. An example of convergence and mixing diagnostics for the 2010 Senegal DHS is shown in the Additional file 5: Figure S1.

Figure 1b shows a scatterplot of modelled ICC estimate against the mean model-based prevalence for the three indicators in the 20 surveys. Figure 1c shows a scatterplot of ICC by indicator against the modelled estimates of ESS with an associated Bayesian credible interval. The range of ICC was between 0 and 0.05 for the three indicators, and was consistent with the expected value for population studies [30, 31]. Zero ICC indicated no correlation of responses between and within the cluster. This was the case, for example, for malaria prevalence in Tanzania, Senegal, and Rwanda (median *ρ* 0 (95% CrI [0–0])). The values of ICC for the indicator, *ρ* = 0.01 or *ρ* = 0.02, suggest that the within-cluster variance was greater than the between-cluster variance. The larger ICC values (*ρ* > 0.02) were associated with a larger prevalence. The effect of ICC was also reflected in the survey design effect which was close to 1.0 (median *deff* 1.01 [1.0–1.01] when *ρ* = 0), for example, for malaria prevalence in the 2010–2011 Rwanda DHS, and in the 2010–2011 and 2012–2013 Senegal DHS surveys. A design effect close to one suggests similarity to simple random sampling. The *deff* was also larger for the larger estimates of ICC (*ρ* > 0.04 and *deff* > 2.0), for example, for fever prevalence in the 2011 Liberia MIS (*deff* 2.52 [2.12–3.02]), or, ITN use in the 2010 Kenya MIS (*deff* 2.40 [2.07–2.77]).

**Fig. 2:**
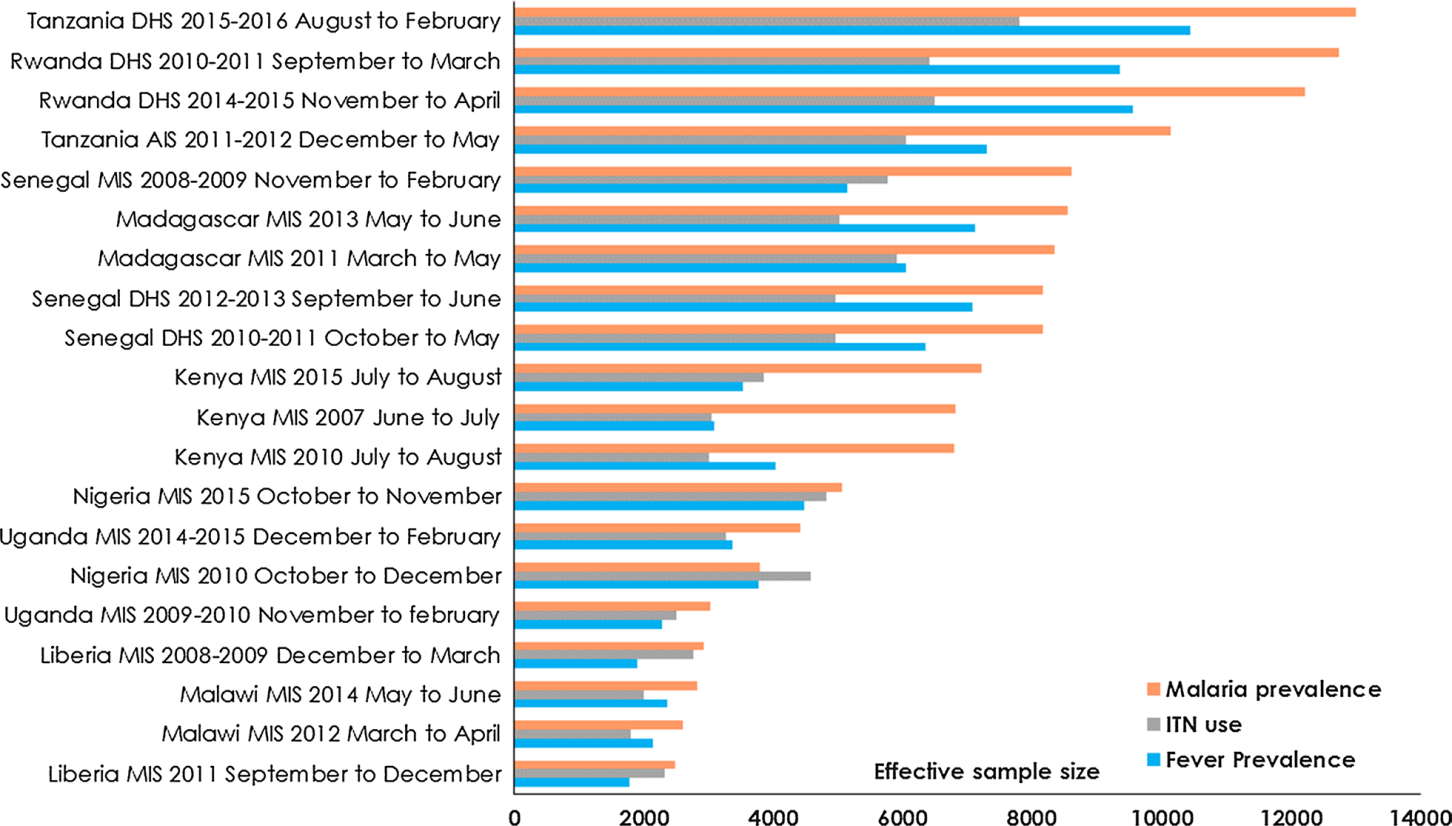
Ranking of country-level estimated effective sample size (ESS) based on RDT positivity (prevalence of malaria) from Bayesian modelling. The countries (y-axis) has been ordered based on RDT positivity from low to high prevalence (Liberia 2011)

Figure 2 shows the ranking for each survey independently by indicator and by prevalence for the modelled ESS. Note that these surveys and countries are assessed independently rather than a direct comparison. The combination of the *deff* and the ICC resulted in mixed results in terms of the modelled estimated effective sample sizes for prevalence of fever, malaria and for ITN use by survey. For some indicators, the ESS was higher while in other indicators lower *deff* (less sampling complexity) and ESS were estimated (mostly for fever and ITN use) (Additional file 6: Figure S2). There was a change in ESS across all the surveys by indicator. For example, in the 2014–2015 Rwanda DHS the estimated ESS for malaria prevalence increased by 54.08% [50.67–56.47] (median ESS of children 0–4 years 12,220 [11,950–12,410]) while in the Tanzania 2015–2016 DHS the ESS increased by 19.26% [18.15–20.26] (ESS 13,000 [12,880–13,110]). The largest percentage increase in the sample size requirement for malaria prevalence based on surveys conducted in the last 5 years was for the 2015 Kenya MIS (by 77.65% [74.72–79.37]; ESS 7129 [7077–7164]). In three countries (Nigeria, Liberia, and Uganda) the ESS was lower than that of the survey sample for children of 0–4 years for all three indicators.

## Discussion

Using malaria indicators as an example, this study showed that variability at cluster level has an impact on the desired sample size for the indicator. On the one hand, the requirement for large sample size to support intervention monitoring reduces with the increasing use of interventions, but on the other hand the sample size increases with declining prevalence (of the indicator). At very low prevalence, variability within clusters was smaller, and the results suggest that large sample sizes are required at this low prevalence especially for blood tests compared to intervention use (ITN use). This suggests defining sample sizes for malaria indicator surveys to increase the precision of detecting prevalence. Comparison between the actual sampled numbers of children aged 0–4 years in the most recent surveys and the estimated effective sample sizes for RDTs showed a deficit in the actual sample size of up to 77.65% [74.72–79.37] for the 2015 Kenya MIS, 25.88% [15.25–35.26] for the 2014 Malawi MIS, 53.3% [54.08–56.47] for the 2014–2015 Rwanda DHS, and by 19.26% [18.15–20.26] for the 2015-to-2016 Tanzania DHS. Smaller required sample sizes (when compared to the actual national sample) were estimated across the three indicators in only Senegal, Liberia, Nigeria, and Uganda suggesting a sub-national relevance of estimates for surveys in these countries.

This study quantified sample size requirements for three malaria-related indicators in recent household surveys. Although such an assessment could be extended to other indicators, it is important to establish the relevance and basis of such an undertaking. Findings here suggest that it may be advantageous to optimize malaria surveys using biomarkers as reference variables. Such an approach could improve precision requirements for other indicators included in these surveys, for example fever prevalence and use of ITNs in children aged 0–4 years, as estimated in these nine study countries with varying transmission. This may also have an added value in surveys where the initial reference sampling indicators were based on prevalence of interventions (for example, the 2007 Kenya MIS [32]), thereby contributing to reduced precision for biomarkers. Biomarkers were introduced in these nationally representative surveys in 2006 and this study highlighted the requirement for larger sample sizes for malaria blood testing in recent surveys. Such biomarkers have the advantage of being an objective measure of health compared to self-reported conditions [33].

There are additional challenges for sub-national variation in malaria prevalence. First, the national malaria programmes face the challenge of expanding future malaria focussed cross-sectional surveys as the malaria burden declines without an increase in resources. For example, in the recent 2015 Kenya MIS, the estimates of intra-class correlation and the design effect suggest that much larger sample sizes may be required for the parasitaemia indicator compared to past survey designs predating the introduction of this RDT-based biomarker. Under conditions of pre-elimination (i.e., parasite prevalence < 1%), the use of surveillance through combined active and passive case detection is already encouraged by the current policy [34, 35]. Thus, there is a need to design cost-effective ways of focussed cross-sectional surveys in sub-national areas experiencing stable malaria transmission with routine surveillance in regions with low-unstable transmission to meet future monitoring and evaluation needs.

While variation between the clusters was observed, the individual responses were correlated within clusters due to similarities in geographic and contextual characteristics. Estimates of intra-class correlation are invariant to scale and, therefore, subsequent estimates of sample sizes require estimates of associated uncertainty as conducted in this study. In general, these estimates have an important utility in interpreting the findings from recent surveys, thus, increasing our understanding of current policy as seen in other health studies [19]. For example, ICC has direct relevance to the disbursement of health interventions. Universal coverage for the entire at-risk population [36] should be emphasized when there is a relatively low degree of clustering [37, 38]. When the ICC is large, a combination of universal and population-based targeting (e.g. households in the low income category or high risk groups) in these settings may be beneficial [39]. In addition, ICC is useful in the planning of future surveys. While the size of survey (i.e., number of clusters) is constrained by funding, estimates of ICC may also inform decisions around which indicators are prioritized. ICC decreases with increasing sample size (Fig. 1c) and larger estimates of ICC amplify the design effect. The design effect is less comparable across countries because it is highly dependent on survey complexity. The design effect is also directly proportional to the average household size and the sampling error, being larger in countries such as Uganda where household size in rural areas was 5.1 compared to 4.1 in urban areas and Nigeria (5.1 and 4.6 for urban and rural areas, respectively).

Estimates for fever were not restricted to children aged 0–4 years with a positive RDT test. Although the proportion (or prevalence) of malaria-related fever decreases with declining transmission [40, 41], some fever cases are still treated presumptively in many settings due to several logistical and operational challenges including the availability of diagnostics, and patient or clinician behaviour [42–44]. Consequently, without the use of parasitological testing, fever cases are undistinguishable from other childhood infections such as respiratory tract infections [45, 46]. In addition, limitations around individual perception of fever, the possibility of multiple episodes of fever within the 14-day window, and recall bias remain [47]. Thus, the current national cluster-sample surveys may underestimate fever prevalence if multiple episodes with varying duration occur within the 14 days preceding the survey. In addition, there are possible limitations of not mimicking the practical realities of these surveys in the Bayesian modelling framework (including cost, and the quality of data collection during fieldwork or non-sampling errors) [48, 49]. While budget plays a role at sample selection stage in addition to other factors such as implementing agency, the aim was to investigate the optimal sample sizes independent of costs in varying transmission intensity settings. The DHS survey, for example, requires a longer fieldwork period (> 6 months) due to the expansive nature of the questionnaire, which presents additional seasonal challenges compared to the MIS (maximum 4 months). Lastly, it is important to note that the objective of the study was not to examine sub-national or fine-scale spatial variation (spatial and temporal heterogeneities) in clustering and prevalence of these indicators, nor to quantify the determinants or drivers of prevalence in the current survey data. Such analysis is carried out as part of mapping studies [50–52], and in studies on the role of environmental drivers in quantifying risk [53–55]. Sub-national precision estimation could, however, be explored by future surveys aiming for more localized survey designs. However, it will be beneficial to first evaluate the costs and benefits of such an adaptation.

## Conclusion

Surveys are often used to provide timely information at a national level. In practice, however, there is usually a trade-off between providing high precision and resource availability at a national level. Firstly, this study highlights the importance of defining indicator-relevant sample sizes, and using biomarkers as reference sampling indicators to achieve the required precision in the current nationally representative surveys. Secondly, the study highlighted that obtaining valid and reliable high quality data for monitoring biomarkers will require expanding current surveys especially in the context of declining prevalence [56]. A major impediment to achieving this, however, is the degree and size of funding allocated to these surveys. The DHS and the Multiple Indicator Cluster Surveys include many more variables and biomarkers such as HIV testing. Thus, while an extended analysis on variability at cluster level in other indicators will also be beneficial, new approaches are required to increase the validity and precision of current surveys including links between household surveys and the administrative level health information systems data.

## Additional files

**Additional file 1.** An extended description of methodology on Bayesian modelling.

**Additional file 2: Table S1.** Data on prevalence of fever indicator from nationally representative household surveys (the demographic health survey (DHS), the malaria indicator survey (MIS) and the HIV/AIDS and malaria indicator survey). Data represents 403,197 children aged 0-4 years in 5,839 clusters and includes surveys where all the three indicators were simultaneously collected at the household level and in countries with more than two surveys. The mean, median and 95% credible intervals of the marginal posterior distribution from the Bayesian analysis is presented for measures of effectiveness: intra-class correlation coefficient (ICC), the estimated design effect (*hdeff*) and the effective sample size (ESS). The absolute bias is the difference in the means between the simple spatial random sample and the Bayesian model estimate. ICC = Intra-class Correlation Coefficient, ESS = Effective sample size; CrI Bayesian Credible Interval.

**Additional file 3: Table S2.** Data on the use of insecticide treated bed nets (ITNs) from nationally representative household surveys (the demographic health survey (DHS), the malaria indicator survey (MIS) and the HIV/AIDS and malaria indicator survey). Data represents 403,197 children aged 0-4 years in 5,839 clusters and includes surveys where all the three indicators were simultaneously collected at the household level and in countries with more than two surveys. The mean, median and 95% credible intervals of the marginal posterior distribution from the Bayesian analysis is presented for measures of effectiveness: intra-class correlation coefficient (ICC), the estimated design effect (*hdeff*) and the effective sample size (ESS). The absolute bias is the difference in the means between the simple spatial random sample and the Bayesian model estimate. ICC = Intra-class Correlation Coefficient, ESS = Effective sample size; CrI Bayesian Credible Interval.

**Additional file 4: Table S3.** Comparison of absolute bias data for Kenya and Rwanda where the spatial random sample represented 20% (Sample 1), 30% (Sample 2) and 40% (Sample 3) hold out set of the clusters. Absolute bias is the difference between the mean from simple random sampling and that generated after MCMC 50,000 iteration in the different cluster-sample.

**Additional file 5: Figure S1.** Convergence diagnostic: Example of trace plots extracted for malaria prevalence survey parameters in the 2010 Senegal DHS for the monitored parameters over the duration of model run.

**Additional file 6: Figure S2.** Country-level comparison between the actual sample size and the estimated effective sample size (ESS) based on the median form Bayesian modelling for **a**) malaria prevalence, **b**) fever prevalence, and **c**) use of ITNs in children under the age of five years.
