# Supplementary material for: Malaria prevalence metrics in low- and middle-income countries: an assessment of precision in nationally-representative surveys
Source: Malar J. 2017 Nov 21;16:475. doi: 10.1186/s12936-017-2127-y (PMC5697056; doi:10.1186/s12936-017-2127-y)
Supplement: Supplementary file 1 — Additional file 1. An extended description of methodology on Bayesian modelling. [file 12936_2017_2127_MOESM1_ESM.docx]

**Additional file 1**

**Malaria prevalence metrics in low- and middle-income countries: an assessment of precision in nationally-representative surveys**

Victor A. Alegana^1, 2*^, Jim Wright^1^, Claudio Bosco^1,2^, Emelda Okiro^5^, Peter M. Atkinson^1,3,4^, Robert Snow^5,6^, Andrew J. Tatem^1,2^, Abdisalan M. Noor^5,6,7^

**1.0 General Bayesian model framework for optimal sample size estimation**

Individual dichotomous response probabilities were modelled assuming a Binomial distribution for individual $i$response in cluster$j$. Thus, $y_{ij}\sim Bin(n,\pi_{i})$with expectation $\pi$and variance $\mathrm{var}(\pi_{i})$. Under a logistic hierarchical Bayesian model, [1, 2]

$$\log it\left(\pi_{i}\right)=\alpha+\beta x_{i}+u_{j}+e_{ij};\begin{matrix} & \end{matrix}e_{ij}\sim N(0,\sigma_{e}^{2}),\begin{matrix} & \end{matrix}u_{i}\sim N(0,\sigma_{u}^{2})$$

Where $i\begin{matrix} (i=1,,,,,n) \end{matrix}$individuals in cluster $j\begin{matrix} (j=1,,,,,k) \end{matrix}$with $u_{j}$allowing for estimation of between cluster variation, $\alpha$as intercept, and $\beta$coefficient estimate for centred age (centring based on the mean) with stratification for cluster urbanicity or rurality. A Bayesian specification [3] was adopted to specify prior distributions [4, 5] for unknown parameters $\alpha$,$\beta$,$\sigma_{u}^{2}$ and $\sigma_{e}^{2}$. We used vague priors for regression parameters $\alpha$and $\beta$, generally$N(0,10)$. An experimentation to modify $\sigma_{e}^{2}$and $\sigma_{u}^{2}$with $\sigma^{2}/weight$was considered but generally viewed as a modification of precision on variance parameter. Hence more general normal truncated priors were considered $N(0,1)I(0,\infty)$where$I$is and indicator function. These precision parameters priors varied by survey but were in general weakly informative and constrained to be positive (e.g.$N(0,0.0001)I(0,\infty)$), with the later estimated based on national estimate of the mean and variance of the variable. Gibbs sampling was then implemented to sample from posterior distribution of parameters providing a posterior distribution of the Intra-class Correlation Coefficient (ICC) $\left(\rho\right)$ [6] and 95% Bayesian Credible Interval (CI):

$$\rho=\frac{\sigma_{u}^{2}}{\sigma_{u}^{2}+\sigma_{e}^{2}}$$

The ICC does is independent of number of clusters or cluster size. Thus is comparable between countries. It related to the survey design effect via:

$$hdeff=\text{1 + }\rho\text{*(m-1)}$$

with $m$specified as average number of households in the cluster for each survey and estimates of $hdeff$were used to derive the effective sample size (ESS). Model deviance as part of Deviance Information Criterion (DIC) was monitored along with other model parameters implemented in MCMC with two chains. The DIC summarises model fit based on a combination of model deviance and complexity (effective number of parameters) [7, 8]. This is defined as $DIC=\bar{D}+pD$where $\bar{D}=E_{\theta|y}[D]$is the mean deviance for $D=-2\times\log\left\{P\left(y|\theta\right)\right\}$.

**2.0 Optimising surveys based on cost**

Most current national representative surveys optimise the final sample size based on funding or budget allocation. Because of the two-stage design, household listing is usually conducted after selection of clusters at the first stage. The overall sample size is a function of ICC and cost. The overall cost ratio (i.e. the cost of surveying a cluster including household listing activities to the cost of interviewing an individual) varies between countries. Thus, high costs of travel between clusters increased the overall cost ratio and a strategy is adopted to conduct fewer but large clusters (in terms of number of households) to meet sampling targets. In general, the cost is calculated as; [9]

$$C_{T}=C_{1}n+C_{2}nm$$

where $C_{T}$is the total cost excluding fixed cost usually subtracted from total cost, $C_{1}$is the unit cost per cluster for $n$number of clusters, while $C_{2}$is unit cost of interviewing an individual for $m$total sample size. Estimating the sample size based on cost using equation from; [10]

$$\begin{matrix} m_{ess}=\sqrt{\frac{1-\rho}{\rho}}c_{r} \\ n_{t}=\frac{C_{T}}{c_{1}+c_{2}m_{ess}} \end{matrix}$$

where $m_{ess}$is the selected total sample size; $n_{t}$is total number of clusters, $\rho$is the intra-class correlation estimated from theoretical variance or standard error assumption prior to the survey, and $c_{r}=c_{1}/c_{2}$is the cost ratio. Thus, increasing homogeneity within the cluster (i.e. increasing$\rho$) suggest selecting fewer households within the cluster but increasing the overall number of cluster and cost of survey.

**References**

1. Groenewald PCN, Mokgatlhe L: Bayesian computation for logistic regression. Comput. Stat. Data Anal. 2005; 48:857-68.

2. Wagner H, Tüchler R: Bayesian estimation of random effects models for multivariate responses of mixed data. Comput. Stat. Data Anal. 2010, 54:1206-18.

3. Spiegelhalter DJ, Abrams KR, Myles JP: An Overview of the Bayesian Approach. In Bayesian Approaches to Clinical Trials and Health-Care Evaluation. John Wiley & Sons, Ltd. 2004. p. 49-120

4. Spiegelhalter DJ, Abrams KR, Myles JP: Prior Distributions. In Bayesian Approaches to Clinical Trials and Health-Care Evaluation. John Wiley & Sons, Ltd. 2004. p. 139-80

5. Gelman A (Ed.). Prior distributions. Chichester: John Wiley & Sons, Ltd. 2002.

6. Turner RM, Omar RZ, Thompson SG: Constructing intervals for the intracluster correlation coefficient using Bayesian modelling, and application in cluster randomized trials. Stat Med. 2006; 25:1443-56.

7. Spiegelhalter DJ, Best NG, Carlin BP, Van Der Linde A: Bayesian measures of model complexity and fit. Journal of the Royal Statistical Society: Series B (Statistical Methodology). 2002; 64:583-639.

8. Plummer M: Penalized loss functions for Bayesian model comparison. Biostatistics. 2008; 9:523-39.

9. ICF International: Demographic and Health Survey Sampling and Household Listing Manual. Calverton, Maryland, U.S.A: ICF International. 2012. <https://dhsprogram.com/pubs/pdf/DHSM4/DHS6_Sampling_Manual_Sept2012_DHSM4.pdf>. Accessed 29 Jan 2017.

10. Cochran WG: Sampling techniques. New York: John Wiley & Sons Inc. 1977.
