# Supplementary material for: Malaria prevalence metrics in low- and middle-income countries: an assessment of precision in nationally-representative surveys
Source: Malar J. 2017 Nov 21;16:475. doi: 10.1186/s12936-017-2127-y (PMC5697056; doi:10.1186/s12936-017-2127-y)
Supplement: Supplementary file 3 — Additional file 3: Table S2. Data on the use of insecticide treated bed nets (ITNs) from nationally representative household surveys (the demographic health survey (DHS), the malaria indicator survey (MIS) and the HIV/AIDS and malaria indicator survey). Data represents 403,197 children aged 0-4 years in 5,839 clusters and includes surveys where all the three indicators were simultaneously collected at the household level and in countries with more than two surveys. The mean, median and 95% credible intervals of the marginal posterior distribution from the Bayesian analysis is presented for measures of effectiveness: intra-class correlation coefficient (ICC), the estimated design effect (hdeff) and the effective sample size (ESS). The absolute bias is the difference in the means between the simple spatial random sample and the Bayesian model estimate. ICC = Intra-class Correlation Coefficient, ESS = Effective sample size; CrI Bayesian Credible Interval. [file 12936_2017_2127_MOESM3_ESM.docx]

**Table S2**: Data on the use of insecticide treated bed nets (ITNs) from nationally representative household surveys (the demographic health survey (DHS), the malaria indicator survey (MIS) and the HIV/AIDS and malaria indicator survey). Data represents 403,197 children aged 0-4 years in 5,839 clusters and includes surveys where all the three indicators were simultaneously collected at the household level and in countries with more than two surveys. The mean, median and 95% credible intervals of the marginal posterior distribution from the Bayesian analysis is presented for measures of effectiveness: intra-class correlation coefficient (ICC), the estimated design effect (*hdeff*) and the effective sample size (ESS). The absolute bias is the difference in the means between the simple spatial random sample and the Bayesian model estimate. ICC = Intra-class Correlation Coefficient, ESS = Effective sample size; CrI Bayesian Credible Interval.

| Country | Survey, year, and months | Number of surveys clusters (Number missing geographic coordinates) | Number of children under five | Simple spatial random sample (mean proportion) | Bias | Simulated proportion. Mean ; median; (95% CrI) | Intra-class correlation coefficient (ICC). Mean ; median; (95% CrI) | Design Effect. Mean (*hdeff*); median; (95% CrI) | Effective sample size (ESS). Mean ; median; (95% CrI) | Percentage increase or decrease (95% CrI) in ESS compared to actual survey sample. |
| --- | --- | --- | --- | --- | --- | --- | --- | --- | --- | --- |
| Kenya | MIS 2007 June to July | 200 (1) | 3,423 | 0.48 | -0.01 | 0.49; 0.49 (0.47-0.51) | 0.04 ;0.04 (0.03-0.05) | 2.37; 2.36 (2.02-2.77) | 3060; 3052 (2600-3558) | -10.84 (-24.04 - 3.94) |
| Kenya | MIS 2010 July to August | 240 (8) | 5,104 | 0.58 | 0.01 | 0.57; 0.57 (0.55-0.59) | 0.05 ;0.05 (0.04-0.06) | 2.4; 2.4 (2.07-2.77) | 3016; 3005 (2595-3481) | -41.12 (-49.16 - -31.8) |
| Kenya | MIS 2015 July to August | 246 (0) | 4,063 | 0.56 | 0.01 | 0.55; 0.55 (0.54-0.57) | 0.03 ;0.03 (0.02-0.04) | 1.92; 1.91 (1.7-2.16) | 3865; 3857 (3417-4347) | -5.07 (-15.9 - 6.99) |
| Liberia | MIS 2008-2009 December to March | 150 (0) | 4,611 | 0.30 | 0.01 | 0.3; 0.3 (0.28-0.31) | 0.02 ;0.02 (0.02-0.03) | 1.63; 1.63 (1.45-1.85) | 2771; 2770 (2432-3113) | -39.93 (-47.26 - -32.49) |
| Liberia | MIS 2011 September to December | 150 (0) | 3,692 | 0.38 | -0.01 | 0.38; 0.38 (0.36-0.4) | 0.03 ;0.03 (0.02-0.04) | 1.94; 1.93 (1.67-2.26) | 2331; 2327 (1989-2690) | -36.97 (-46.13 - -27.14) |
| Madagascar | MIS 2011 March to May | 268 (1) | 7,138 | 0.76 | -0.01 | 0.77; 0.77 (0.76-0.78) | 0.01 ;0.01 (0.01-0.02) | 1.45; 1.45 (1.34-1.58) | 5909; 5907 (5415-6386) | -17.25 (-24.14 - -10.54) |
| Madagascar | MIS 2013 May to June | 274 (0) | 6,288 | 0.54 | -0.04 | 0.58; 0.58 (0.57-0.6) | 0.02 ;0.02 (0.02-0.03) | 1.75; 1.75 (1.58-1.94) | 5027; 5020 (4529-5542) | -20.17 (-27.97 - -11.86) |
| Malawi | MIS 2012 March to April | 140 (0) | 2,436 | 0.58 | -0.01 | 0.6; 0.6 (0.57-0.62) | 0.04 ;0.04 (0.03-0.05) | 1.95; 1.94 (1.67-2.28) | 1803; 1803 (1538-2094) | -25.99 (-36.86 - -14.04) |
| Malawi | MIS 2014 May to June | 140 (0) | 2,249 | 0.70 | -0.04 | 0.74; 0.74 (0.72-0.76) | 0.03 ;0.03 (0.02-0.04) | 1.76; 1.75 (1.52-2.06) | 2000; 1999 (1698-2305) | -11.12 (-24.5 - 2.49) |
| Nigeria | MIS 2010 October to December | 239 (0) | 4,950 | 0.31 | 0.00 | 0.3; 0.3 (0.29-0.31) | 0.01 ;0.01 (0.01-0.02) | 1.36; 1.36 (1.27-1.47) | 4575; 4581 (4222-4908) | -7.45 (-14.71 - -0.85) |
| Nigeria | MIS 2015 October to November | 326 (4) | 7,016 | 0.42 | 0.00 | 0.41; 0.41 (0.4-0.42) | 0.03 ;0.03 (0.02-0.04) | 1.7; 1.69 (1.56-1.85) | 4816; 4817 (4394-5230) | -31.34 (-37.37 - -25.46) |
| Rwanda | DHS 2010-2011 September to March | 492 (0) | 8,963 | 0.70 | 0.01 | 0.69; 0.69 (0.68-0.7) | 0.04 ;0.04 (0.03-0.05) | 2; 2 (1.84-2.17) | 6418; 6412 (5890-6962) | -28.46 (-34.29 - -22.33) |
| Rwanda | DHS 2014-2015 November to April | 492 (0) | 7,931 | 0.67 | 0.00 | 0.67; 0.67 (0.66-0.68) | 0.04 ;0.04 (0.03-0.05) | 1.97; 1.97 (1.83-2.15) | 6494; 6494 (5945-7005) | -18.12 (-25.04 - -11.68) |
| Senegal | MIS 2008-2009 November to February | 320 (2) | 16,156 | 0.33 | 0.02 | 0.31; 0.31 (0.3-0.32) | 0.02 ;0.02 (0.02-0.03) | 1.67; 1.66 (1.53-1.81) | 5772; 5772 (5301-6257) | -64.27 (-67.19 - -61.27) |
| Senegal | DHS 2010-2011 October to May | 391 (6) | 13,334 | 0.45 | 0.00 | 0.44; 0.44 (0.44-0.45) | 0.03 ;0.03 (0.03-0.04) | 1.66; 1.66 (1.54-1.79) | 4956; 4954 (4595-5334) | -62.85 (-65.54 - -60) |
| Senegal | DHS 2012-2013 September to June | 200 (0) | 7,413 | 0.50 | 0.01 | 0.48; 0.48 (0.47-0.5) | 0.03 ;0.03 (0.02-0.04) | 1.66; 1.65 (1.49-1.83) | 4973; 4966 (4484-5499) | -33.01 (-39.51 - -25.82) |
| Tanzania | HIV/AIDS and MIS 2011-2012 December to May | 583 (10) | 9,319 | 0.69 | 0.01 | 0.69; 0.69 (0.68-0.7) | 0.04 ;0.03 (0.03-0.04) | 1.74; 1.73 (1.62-1.86) | 6054; 6055 (5640-6474) | -35.03 (-39.48 - -30.53) |
| Tanzania | DHS 2015-2016 August to February | 608 (0) | 10,901 | 0.50 | -0.01 | 0.51; 0.51 (0.5-0.52) | 0.03 ;0.03 (0.03-0.04) | 1.72; 1.72 (1.61-1.83) | 7804; 7800 (7315-8301) | -28.45 (-32.9 - -23.85) |
| Uganda | MIS 2009-2010 November to February | 170 (0) | 4,202 | 0.33 | -0.01 | 0.34; 0.34 (0.33-0.36) | 0.03 ;0.03 (0.03-0.04) | 1.9; 1.9 (1.68-2.17) | 2512; 2509 (2196-2834) | -40.29 (-47.74 - -32.56) |
| Uganda | MIS 2014-2015 December to February | 210 (2) | 5,210 | 0.74 | 0.00 | 0.75; 0.75 (0.74-0.76) | 0.03 ;0.03 (0.02-0.04) | 1.8; 1.8 (1.61-2.02) | 3273; 3274 (2907-3664) | -37.16 (-44.2 - -29.67) |
