# Supplementary material for: Malaria prevalence metrics in low- and middle-income countries: an assessment of precision in nationally-representative surveys
Source: Malar J. 2017 Nov 21;16:475. doi: 10.1186/s12936-017-2127-y (PMC5697056; doi:10.1186/s12936-017-2127-y)
Supplement: Supplementary file 4 — Additional file 4: Table S3. Comparison of absolute bias data for Kenya and Rwanda where the spatial random sample represented 20% (Sample 1), 30% (Sample 2) and 40% (Sample 3) hold out set of the clusters. Absolute bias is the difference between the mean from simple random sampling and that generated after MCMC 50,000 iteration in the different cluster-sample. [file 12936_2017_2127_MOESM4_ESM.docx]

**Table S3:** Comparison of absolute bias data for Kenya and Rwanda where the spatial random sample represented 20% (Sample 1), 30% (Sample 2) and 40% (Sample 3) hold out set of the clusters. Absolute bias is the difference between the mean from simple random sampling and that generated after MCMC 50,000 iteration in the different cluster-sample.

| **Country, Survey** | **Indicator** | **Sample** | **SRS mean (proportion)** | **Bias** | **Estimated mean, SD, median (95% CI)** |
| --- | --- | --- | --- | --- | --- |
| Kenya 2007 MIS | Fever | 1 | 0.32 | -0.01 | 0.33 ;0.01; 0.33 (0.31-0.34) |
|  | Fever | 2 | 0.32 | -0.01 | 0.33 ;0.01; 0.33 (0.31-0.35) |
|  | Fever | 3 | 0.32 | -0.02 | 0.33 ;0.01; 0.33 (0.31-0.35) |
|  | ITN Use | 1 | 0.48 | -0.01 | 0.49 ;0.01; 0.49 (0.47-0.5) |
|  | ITN Use | 2 | 0.48 | -0.01 | 0.49 ;0.01; 0.49 (0.47-0.51) |
|  | ITN Use | 3 | 0.48 | -0.01 | 0.49 ;0.01; 0.49 (0.47-0.51) |
|  | Malaria prevalence | 1 | 0.07 | -0.01 | 0.08 ;0; 0.08 (0.07-0.09) |
|  | Malaria prevalence | 2 | 0.07 | -0.01 | 0.09 ;0; 0.09 (0.08-0.1) |
|  | Malaria prevalence | 3 | 0.07 | -0.01 | 0.09 ;0.01; 0.09 (0.08-0.1) |
| Kenya 2010 MIS | Fever | 1 | 0.28 | 0.00 | 0.28 ;0.01; 0.28 (0.26-0.3) |
|  | Fever | 2 | 0.28 | 0.00 | 0.28 ;0.01; 0.28 (0.26-0.31) |
|  | Fever | 3 | 0.28 | 0.01 | 0.28 ;0.01; 0.28 (0.25-0.3) |
|  | ITN Use | 1 | 0.58 | 0.00 | 0.57 ;0.01; 0.57 (0.56-0.59) |
|  | ITN Use | 2 | 0.58 | 0.01 | 0.57 ;0.01; 0.57 (0.55-0.59) |
|  | ITN Use | 3 | 0.58 | 0.00 | 0.58 ;0.01; 0.58 (0.56-0.6) |
|  | Malaria prevalence | 1 | 0.14 | 0.00 | 0.13 ;0; 0.13 (0.13-0.14) |
|  | Malaria prevalence | 2 | 0.14 | 0.00 | 0.14 ;0; 0.14 (0.13-0.14) |
|  | Malaria prevalence | 3 | 0.14 | 0.02 | 0.12 ;0; 0.12 (0.11-0.13) |
| Kenya 2015 MIS | Fever | 1 | 0.38 | -0.02 | 0.4 ;0.01; 0.4 (0.38-0.42) |
|  | Fever | 2 | 0.38 | -0.02 | 0.4 ;0.01; 0.4 (0.38-0.41) |
|  | Fever | 3 | 0.38 | -0.03 | 0.41 ;0.01; 0.41 (0.39-0.43) |
|  | ITN Use | 1 | 0.56 | 0.01 | 0.55 ;0.01; 0.55 (0.54-0.57) |
|  | ITN Use | 2 | 0.56 | 0.01 | 0.55 ;0.01; 0.55 (0.54-0.57) |
|  | ITN Use | 3 | 0.56 | 0.01 | 0.55 ;0.01; 0.55 (0.53-0.57) |
|  | Malaria prevalence | 1 | 0.09 | -0.01 | 0.1 ;0; 0.1 (0.09-0.11) |
|  | Malaria prevalence | 2 | 0.09 | -0.01 | 0.1 ;0; 0.1 (0.09-0.11) |
|  | Malaria prevalence | 3 | 0.09 | -0.02 | 0.11 ;0.01; 0.11 (0.1-0.12) |
| Rwanda 2010 DHS | Fever | 1 | 0.16 | -0.01 | 0.17 ;0; 0.17 (0.16-0.18) |
|  | Fever | 2 | 0.16 | -0.01 | 0.17 ;0; 0.17 (0.16-0.18) |
|  | Fever | 3 | 0.16 | -0.01 | 0.17 ;0.01; 0.17 (0.16-0.18) |
|  | ITN Use | 1 | 0.70 | 0.00 | 0.69 ;0.01; 0.69 (0.68-0.7) |
|  | ITN Use | 2 | 0.70 | 0.01 | 0.69 ;0.01; 0.69 (0.68-0.7) |
|  | ITN Use | 3 | 0.70 | 0.01 | 0.69 ;0.01; 0.69 (0.67-0.7) |
|  | Malaria prevalence | 1 | 0.02 | 0.00 | 0.03 ;0; 0.03 (0.02-0.03) |
|  | Malaria prevalence | 2 | 0.02 | 0.00 | 0.02 ;0; 0.02 (0.02-0.03) |
|  | Malaria prevalence | 3 | 0.02 | 0.00 | 0.02 ;0; 0.02 (0.02-0.03) |
| Rwanda 2014 DHS | Fever | 1 | 0.19 | -0.02 | 0.2 ;0; 0.2 (0.19-0.21) |
|  | Fever | 2 | 0.19 | -0.02 | 0.2 ;0.01; 0.2 (0.19-0.21) |
|  | Fever | 3 | 0.19 | -0.01 | 0.2 ;0.01; 0.2 (0.19-0.21) |
|  | ITN Use | 1 | 0.67 | 0.00 | 0.67 ;0.01; 0.67 (0.66-0.68) |
|  | ITN Use | 2 | 0.67 | 0.00 | 0.67 ;0.01; 0.67 (0.66-0.68) |
|  | ITN Use | 3 | 0.67 | 0.00 | 0.67 ;0.01; 0.67 (0.66-0.68) |
|  | Malaria prevalence | 1 | 0.08 | -0.01 | 0.09 ;0; 0.09 (0.08-0.1) |
|  | Malaria prevalence | 2 | 0.08 | -0.01 | 0.08 ;0.01; 0.08 (0.07-0.09) |
|  | Malaria prevalence | 3 | 0.08 | 0.00 | 0.08 ;0.01; 0.08 (0.07-0.09) |
