# Supplementary material for: Malaria prevalence metrics in low- and middle-income countries: an assessment of precision in nationally-representative surveys
Source: Malar J. 2017 Nov 21;16:475. doi: 10.1186/s12936-017-2127-y (PMC5697056; doi:10.1186/s12936-017-2127-y)

**Fig S1**: Convergence diagnostic: Example of trace plots extracted for malaria prevalence survey parameters in the 2010 Senegal DHS for the monitored parameters over the duration of model run.


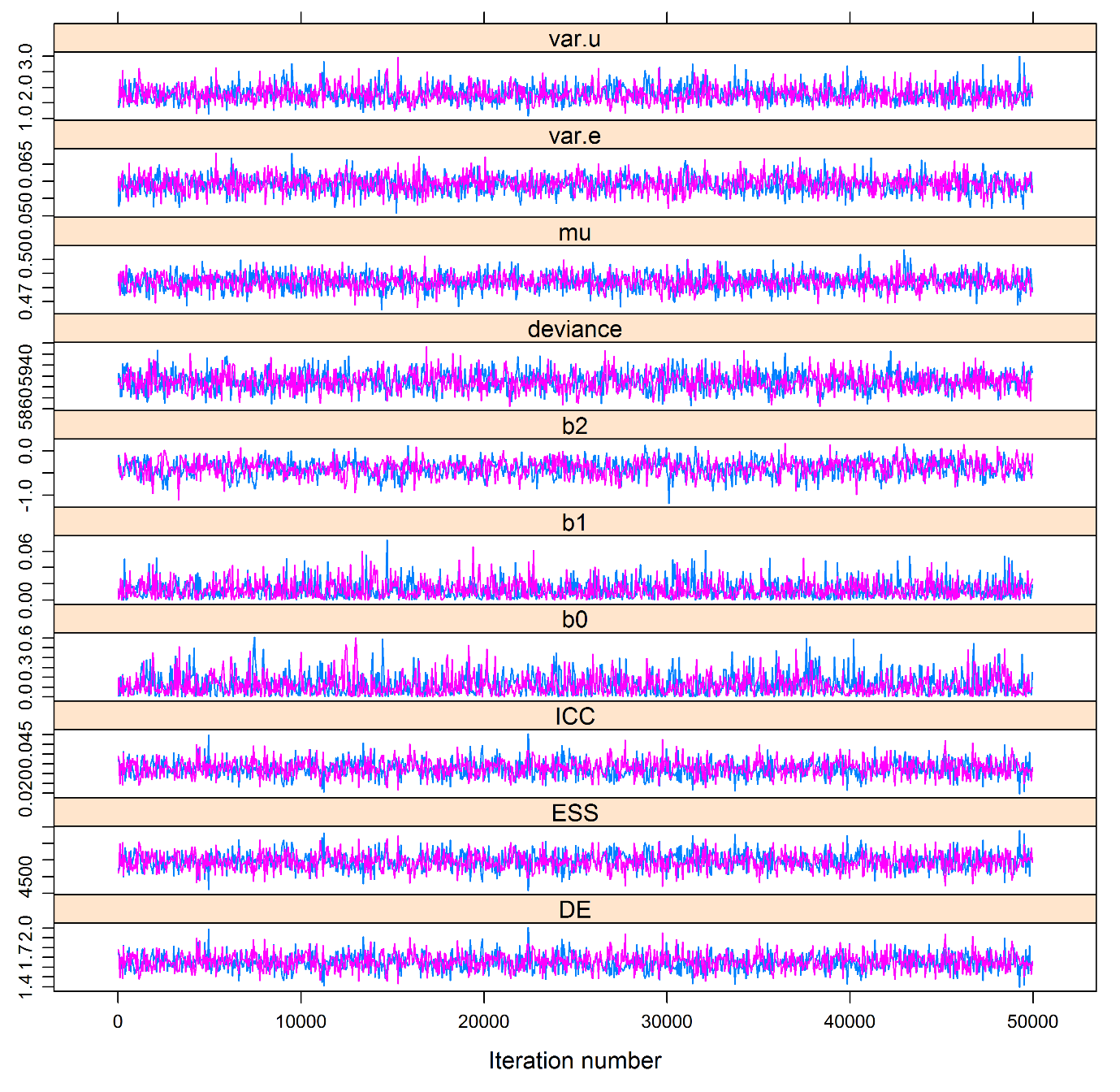

Supplement: Supplementary file 5 — Additional file 5: Figure S1. Convergence diagnostic: Example of trace plots extracted for malaria prevalence survey parameters in the 2010 Senegal DHS for the monitored parameters over the duration of model run. [file 12936_2017_2127_MOESM5_ESM.docx]
