# Supplementary material for: Malaria prevalence metrics in low- and middle-income countries: an assessment of precision in nationally-representative surveys
Source: Malar J. 2017 Nov 21;16:475. doi: 10.1186/s12936-017-2127-y (PMC5697056; doi:10.1186/s12936-017-2127-y)

**Fig S2**: Country-level comparison between the actual sample size and the estimated effective sample size (ESS) based on the median form Bayesian modelling for **a)** malaria prevalence, **b)** fever prevalence, and **c)** use of ITNs in children under the age of five years.


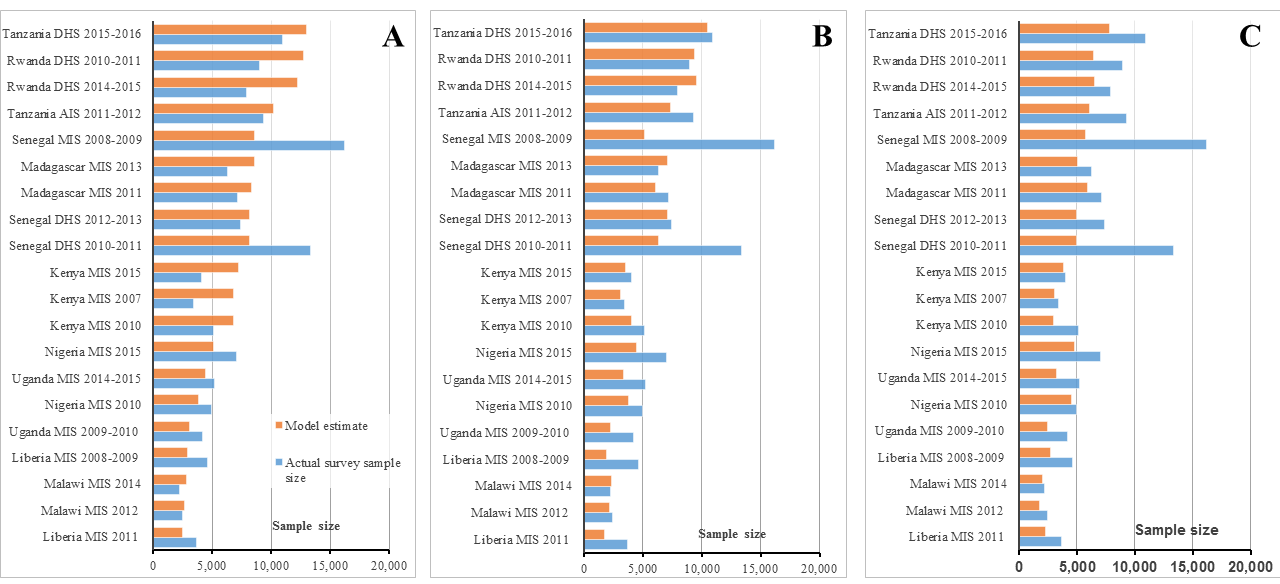

Supplement: Supplementary file 6 — Additional file 6: Figure S2. Country-level comparison between the actual sample size and the estimated effective sample size (ESS) based on the median form Bayesian modelling for a) malaria prevalence, b) fever prevalence, and c) use of ITNs in children under the age of five years. [file 12936_2017_2127_MOESM6_ESM.docx]
